# Evaluation of Mask-Induced Cardiopulmonary Stress: A Randomized Crossover Trial

**DOI:** 10.1001/jamanetworkopen.2023.17023

**Published:** 2023-06-09

**Authors:** Riqiang Bao, Guang Ning, Yingkai Sun, Shijia Pan, Weiqing Wang

**Affiliations:** 1 Department of Endocrine and Metabolic Diseases, Shanghai Institute of Endocrine and Metabolic Diseases, Ruijin Hospital, Shanghai Jiaotong University, School of Medicine, Shanghai, China; 2 Shanghai National Clinical Research Center for Metabolic Diseases, Key Laboratory for Endocrine and Metabolic Diseases of the National Health Commission of the PR China, Shanghai National Center for Translational Medicine, Ruijin Hospital, Shanghai Jiaotong University, School of Medicine, Shanghai, China; 3 Shanghai Digital Medicine Innovation Center, Shanghai, China

## Abstract

This randomized crossover trial evaluates the cardiopulmonary effects of extended use of the N95 mask during daily life.

## Introduction

Face masks have been proven effective in reducing the transmission of COVID-19.^1^ As airborne diseases continue to emerge, mask use is still suggested in public and work spaces as a precautionary measure. In China, mask use remains a highly adopted practice in everyday life.^2^ However, studies on the adverse effects of wearing masks yielded inconsistent conclusions due to short duration of intervention.^3,4,5^ Given that the N95 mask offers the highest level of protection against viruses such as COVID-19, we systematically evaluated the effects of extended use of the N95 mask during daily life.

## Methods

**Figure 1.**
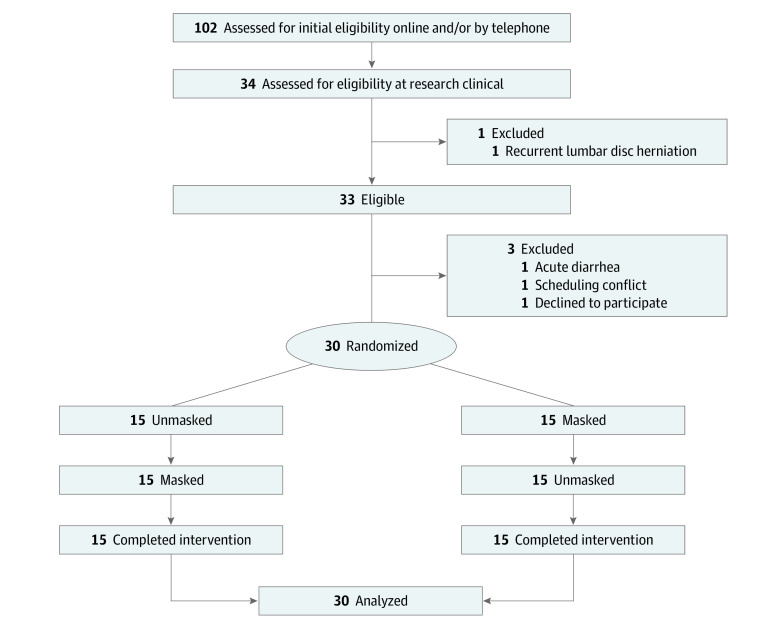
Study Flowchart

This randomized clinical trial included 30 healthy participants between March 7 and August 1, 2022, in Shanghai, China. The trial protocol (Supplement 1) was approved by the review board of Ruijin Hospital affiliated with Shanghai Jiaotong University, and all participants provided written informed consent. This study followed the CONSORT reporting guideline (Figure 1).

The study was conducted in a metabolic chamber to strictly control daily calorie intake and physical activity levels. With the use of stratified randomization, participants were randomly assigned to receive interventions with and without the N95 mask (9132; 3M) for 14 hours (8:00 to 22:00), during which they exercised for 30 minutes in the morning and afternoon using an ergometer at 40% (light intensity) and 20% (very light intensity) of their maximum oxygen consumption levels, respectively. Venous blood samples were taken before and 14 hours after the intervention for blood gas and metabolite analysis (eMethods, eTable, and eFigure in Supplement 2).

A sample size of 30 participants was required, based on our preliminary data of the mean (SD) heart rate between masked (87.5 [3.4] beats/min) and unmasked (85.7 [2.9] beats/min) conditions and to achieve 85% power and a significance level of .05. Analysis was performed on a per-protocol basis. Differences were estimated using a linear mixed-effects model. Where significant, Bonferroni-corrected post hoc tests were performed. Statistical tests were 2-sided. Statistical analyses were conducted using R, version 4.02 (R Group for Statistical Computing).

## Results

**Figure 2.**
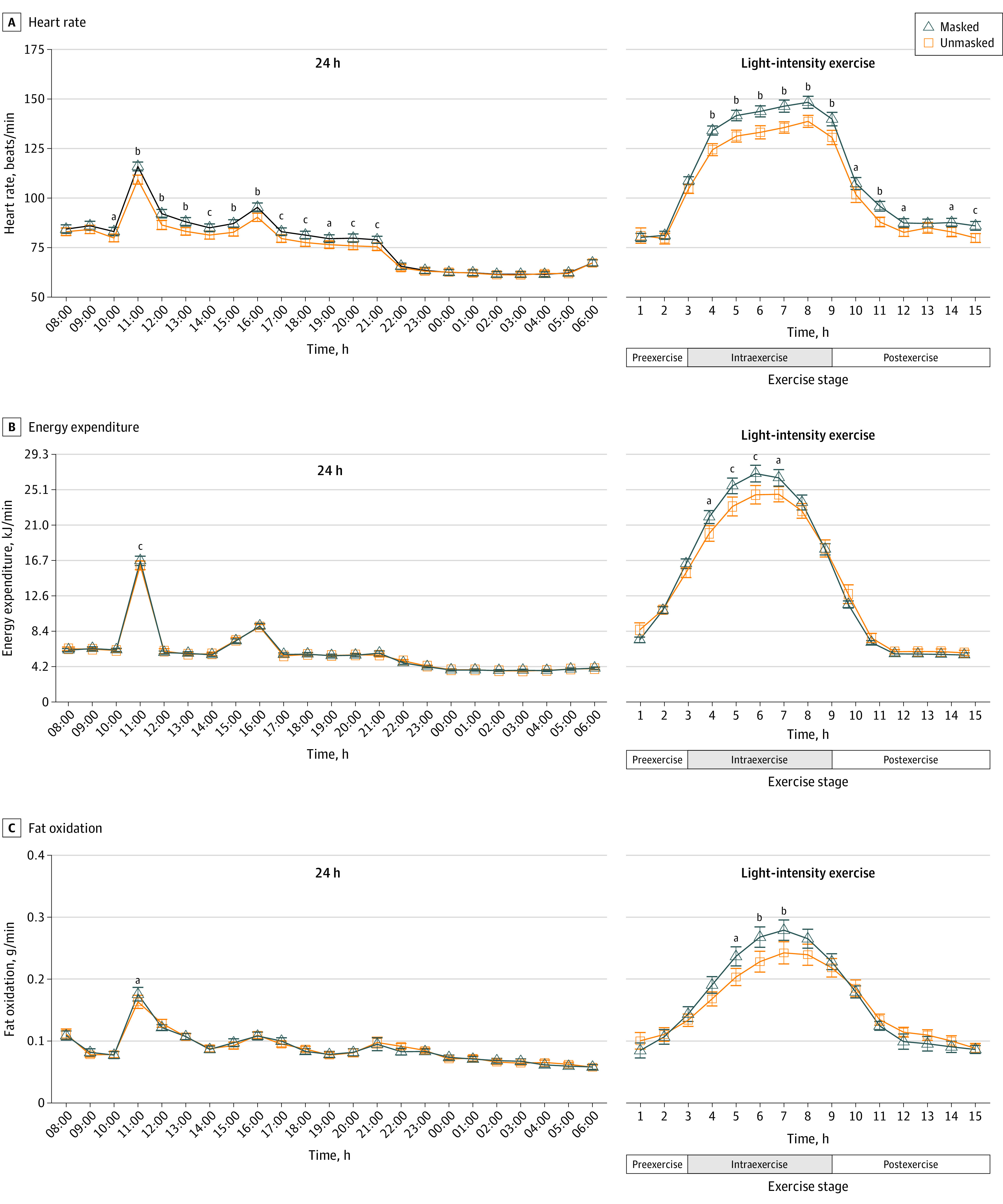
Effects of Wearing an N95 Mask on Physiological and Biochemical Parameters Parameters over 24 hours and light-intensity exercise were compared between unmasked and masked conditions. ^a^*P* < .05. ^b^*P* < .001. ^c^*P* < .01.

Thirty randomized participants (mean [SD] age, 26.1 [2.9] years; 15 women [50%]) completed the study. Wearing the N95 mask resulted in reduced respiration rate and oxygen saturation by pulse oximetry (Spo_2_) within 1 hour, with elevated heart rate (mean change, 3.8 beats/min [95% CI, 2.6-5.1 beats/min]) 2 hours later until mask off at 22:00. During the light-intensity exercise at 11:00, mask-induced cardiopulmonary stress was further increased, as heart rate (mean change, 7.8 beats/min [95% CI, 5.3-10.2 beats/min]) and blood pressure (systolic: mean change, 6.1 mm Hg [95% CI, 0.6-11.5 mm Hg]; diastolic: mean change, 5.0 mm Hg [95% CI, 0.3-9.6 mm Hg]) increased, while respiration rate (mean change, −4.3 breaths/min [95% CI, −6.4 to 2.3 breaths/min]) and Spo_2_ (mean change, −0.66% [95% CI, −1.0% to 0.3%]) decreased. Energy expenditure (mean change, 0.5 kJ [95% CI, 0.2-0.8] kJ) and fat oxidation (mean change, 0.01 g/min [95% CI, −0.01 to 0.03 g/min]) were elevated at 11:00. After the 14-hour masked intervention, venous blood pH decreased, and calculated arterial pH showed a decreasing trend. Metanephrine and normetanephrine levels were increased. Participants also reported increased overall discomfort with the N95 mask (Figure 2).

## Discussion

The findings contribute to existing literature by demonstrating that wearing the N95 mask for 14 hours significantly affected the physiological, biochemical, and perception parameters.^4,5^ The effect was primarily initiated by increased respiratory resistance and subsequent decreased blood oxygen and pH, which contributed to sympathoadrenal system activation and epinephrine as well as norepinephrine secretion elevation. The extra hormones elicited a compensatory increase in heart rate and blood pressure. Although healthy individuals can compensate for this cardiopulmonary overload, other populations, such as elderly individuals, children, and those with cardiopulmonary diseases, may experience compromised compensation. Chronic cardiopulmonary stress may also increase cardiovascular diseases and overall mortality.^6^ However, the study was limited to only 30 young healthy participants in a laboratory setting; further investigation is needed to explore the effects of different masks on various populations in clinical settings.

## Notes

Supplement 1.
Trial Protocol

Supplement 2.
eMethods.
**eFigure.** Study Design **eTable.** Basal Characteristics of Participants
eReference.

Supplement 3.
Data Sharing Statement
