# Supplementary material for: Evaluation of Mask-Induced Cardiopulmonary Stress: A Randomized Crossover Trial
Source: JAMA Netw Open. 2023 Jun 9;6(6):e2317023. doi: 10.1001/jamanetworkopen.2023.17023 (PMC10257095; doi:10.1001/jamanetworkopen.2023.17023)
Supplement: Supplement 1. — Trial Protocol [file jamanetwopen-e2317023-s001.pdf]

Clinical Research Program, Ruijin Hospital, Shanghai Jiao Tong University

**Study Name**	A randomized crossover clinical study to evaluate the effect of N95 masks on human energy metabolism
**Program Number**	
**Principal Investigator**	Prof. Weiqing Wang
**Group leader unit**	Ruijin Hospital, Shanghai Jiaotong University School of Medicine
**Program Start Date**	2022.3
**Program End Date**	2022.10

Catalog

1 Research Summary 3
1.1 Abstract 3
1.2 Schematic diagram of the study 6
2 Research Background 6
2.1 Research significance 6
2.2 Research Background 6
2.3 Expected results of the study 6
2.3.1 Known potential risks. 7
2.3.2 Known potential benefits. 7
2.3.3 Potential risk/benefit assessment 7
3 Principal Investigator Information 7
3.1 Principal investigator's name, qualifications, contact information 7
3.2 Key participants. 7
4 Purpose of the study 7
1. Main purpose. 7
2. Secondary purpose.. 8
5 Study Design 8
5.1 Overall design 8
5.2 Study endpoints 8
5.3 Sample size 8
6 Research Subjects 9
6.1 Selection Criteria. 9
6.2 Exclusion Criteria 9
6.3 Study Subject Recruitment 10
6.4 Methodology of research subject assignment 10
7 Research interventions 10
7.1 Intervention content 10
7.1.1 Intervention method 10
7.1.2 The items and number of clinical and laboratory tests to be performed 10
2. General Information 10
3. Medical history 10
4. Past medication history 11
5. Vital signs, physical examination 11
6. 12-lead electrocardiogram 11
7. Energy metabolism index 11
8. Maximum oxygen consumption measurement 11
9. Laboratory tests 11
10. Retention of biological samples 13

11. Adherence evaluation ... 13
7.2 Measures to reduce bias: randomization and control of confounding factors ... 13
7.3 Adherence judgment ... 13
Screening Period ... 13
Balance period ... 13
Expectations ... 13
7.4 Research Intervention Commitment ... 13
7.5 Research Flow Chart ... 14

8 Study intervention termination and subject termination/withdrawal ... 19
8.1 Study intervention termination ... 19
8.2 Subject termination/withdrawal ... 19

9 Evaluation of study outcomes ... 19
9.1 Primary and secondary efficacy evaluation indicators ... 19
9.2 Safety evaluation indicators ... 20
9.3 Adverse Events and Serious Adverse Events ... 20
9.3.1 Adverse Event (AE) Definition ... 20
9.3.2 Serious Adverse Event (SAE) Definition ... 20
9.3.3 Adverse Event Reporting ... 20
9.3.4 Serious Adverse Event Reporting ... 20

10 Statistical Analysis ... 20
10.1 Analysis of data sets ... 20
10.2 General Method ... 21
10.3 Analysis of primary and secondary study endpoints ... 21
10.4 Baseline descriptive analysis ... 21

11 Supporting Documents and Notes ... 21
11.1 Informed Consent ... 21
11.2 Privacy Protection ... 23
11.3 Specimen and data collection and use ... 23
11.3.1 Experimental Process Management ... 23
11.3.2 Improve observation consistency measures ... 23
11.3.3 Laboratory quality control requirements ... 25
11.3.4 Measures to Ensure Subject Adherence ... 25
11.4 Data processing and record keeping ... 25
11.4.1 Data Collection and Management ... 25
11.4.2 Publishing and data sharing conventions ... 27
11.4.3 Conflict of Interest Statement ... 27

12 Refereces ... 27

13 Annexes ... 27
13.1 Standard operating procedures for sample testing and delivery ... 27

I Research Summary

1.1 Abstract

**Study Name**	A randomized crossover clinical study to evaluate the effect of N95 masks on human energy metabolism
**Research Introduction**	This study is a randomized, self-crossover controlled single-center clinical study. Metabolically healthy volunteers are planned to be recruited for this study. The study is divided into three phases: screening period (within 10 days), equilibration period (1 day), and intervention period (2 days). Screening eligible subjects will be randomly assigned to either the control-intervention group or the intervention-control group in a 1:1 ratio. Each subject will receive 14 hours of continuous N95 mask wear on the day of the intervention. Metabolism and vital signs will be collected during and before and after the intervention.
**Purpose of the study**	• Main purpose.<br>Evaluation of the effects of N95 masks on glucose and lipid metabolism and the cardiovascular system in subjects<br>• Secondary purpose.<br>Evaluation of the effect of wearing N95 masks on sleep quality Evaluation of the effect of wearing N95 masks on respiration and oxygen consumption Evaluation of the effect of wearing N95 masks on endocrine hormones
**Research Subjects**	The study was planned to include 30 healthy volunteers who were rigorously screened (strict laboratory indices, sex ratio, age and BMI range) and randomly assigned to the experimental order in a 1:1 ratio.<br>• Inclusion criteria.<br>1. Healthy people aged 18 - 35 years<br>2. BMI between 18 - 28<br>3. Stable weight with less than ±3% change in the last three months<br>4. Healthy lifestyle habits, eating, sleeping and exercising regularly<br>5. No medication for the last three months<br>6. Fasting blood glucose < 6.1 mmol/L, HbA1c < 6.4%, no abnormal liver or kidney function during the screening period<br>7. No caffeine and alcohol intake habits<br>8. No smoking habit<br>9. Able to sign informed consent forms independently

(continued)

	Exclusion criteria.<br>1. Patients with diabetes, hyperlipidemia, hypertension.<br>2. Other serious organic heart disease, such as congenital heart disease, rheumatic heart disease, hypertrophic or expanded cardiomyopathy, chronic congestive heart failure (NYHA ≥ Class III), or those undergoing interventional therapy or using a cardiac stent, or those with stage III hypertension (systolic blood pressure cannot be controlled below 160 mmHg with three antihypertensive drugs)<br>3. People with hyper- or hypothyroidism.<br>4. treated or untreated organ system tumors (other than localized cutaneous basal cell carcinoma) within the past 5 years, regardless of whether there is evidence of local recurrence or metastasis.<br>5. Gastrointestinal disorders affecting food digestion and absorption (e.g., severe abdominal feces, constipation, irritable bowel syndrome, inflammatory bowel disease, active peptic ulcer, acute cholecystitis, etc.): severe abdominal feces means watery stools twice a day or more for a period of days or more, severe constipation means 2 or fewer bowel movements per week with difficulty in defecation.<br>6. Persons with any mental illness or history of mental illness; persons with epilepsy or on antidepressant medication as part of their anti-madness treatment.<br>7. Persons with severe infections, severe anemia, neutropenia.<br>8. Major surgery (except appendicitis, hernia surgery) within 1 year prior to screening.<br>9. History of previous bowel resection, or other GI surgery (e.g. cholecystectomy) within 1 year prior to screening, or other non-GI surgery within 6 months;<br>10. Use of weight control drugs (including diet pills), hormonal drugs such as corticosteroids (including oral, intramuscular or intravenous systemic, non-digestive or intra-articular) within 3 months prior to screening or planned during the trial.<br>11. Severe hepatic or renal dysfunction, or alanine aminotransferase (ALT) or aspartate aminotransferase (AST) ≥2.5× ULN, serum creatinine > UIN, glomerular filtration rate (eGFR) < 60mL/min/1.73m2 at screening (calculated using the MDRD formula)<br>12. Night shift or jet lag in the 1 month prior to screening<br>13. Antibiotic users in the 3 months prior to screening<br>14. Those who plan to use drugs (including traditional Chinese medicine and proprietary Chinese medicine) to change their body weight within 3 months before screening or during the study period, or those whose body weight fluctuates significantly (≥3kg) during the 3 months before screening.<br>15. Persons with a history of active substance abuse and/or alcoholism (> 40 g/day of alcohol for men and<br>16. For example, 40g of alcohol is about 1000mL of beer (6 degrees), or 500mL of red or yellow wine (12 degrees) for ≥20g/day.<br>17. degree), or 100mL white wine (50 degree))<br>18. Pregnant or lactating women<br>19. Participation in other clinical studies within 3 months prior to screening.<br>20. Any condition deemed by the investigator to be inappropriate for participation in this study.

(continued)

<table>
<tr><td>Research interventions</td><td>

Research interventions :
<u>Intervention day</u>: Wear the N95 mask from 08:00 and keep it on for the rest of the day, except for a short time during meals. Continue to remove until 22:00 on the same day
<u>Control day</u>: no mask, but activities including meals, exercise, and sleep during 24 hours were consistent with the intervention phase
Evaluation metrics :
<u>Validity indicators</u>

Main evaluation indicators
✓ Heart rate
✓ Metabolic rate
✓ Intravenous blood glucose
✓ Glycolipid oxidation amount
Secondary evaluation indicators
✓ Oxygen saturation
✓ Respiratory rate
✓ Blood pressure
✓ Sleep Staging
✓ Endocrine hormones
✓ Blood lipids

<u>Safety indicators</u>
Adverse events, physical examination, vital signs, 12-lead electrocardiogram, laboratory tests (routine blood, urine, blood biochemistry)

</td></tr>
<tr><td>Research Unit</td><td>Principal Investigator and Affiliation: Prof. Weiqing Wang, Ruijin Hospital, Shanghai Jiaotong University School of Medicine</td></tr>
<tr><td>Duration of the study</td><td>Start in March 2022, expected to be completed in June 2022</td></tr>
<tr><td>Subjects' participation time</td><td>Each subject is required to pass a screening period, a stabilization period, and an intervention period for a total of approximately 6 days</td></tr>
</table>

1.2 Schematic diagram of the study

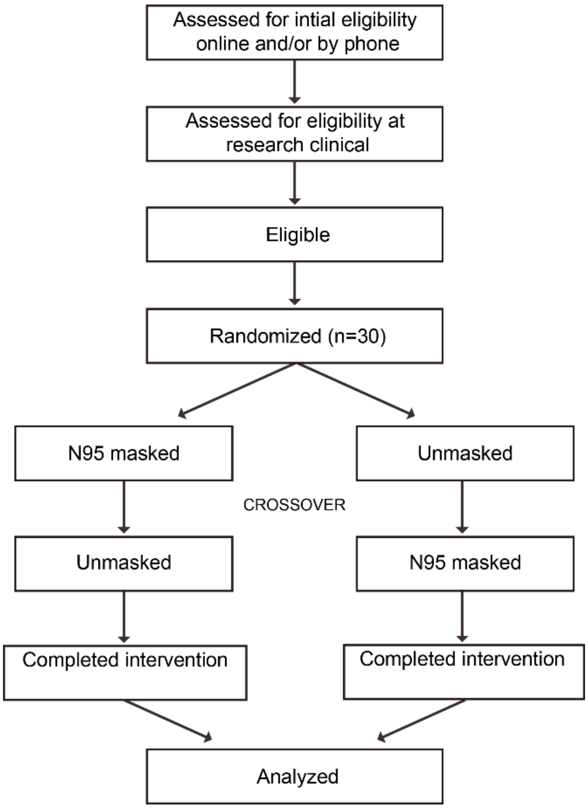

2 Research Background

2.1 Research significance

Since the beginning of the COVID-19 pandemic, N95 masks have been used extensively worldwide in everyday and medical scenarios. To avoid the spread of COVID-19, wearers often wear them for long periods of time. Several studies have now reported a global surge in the incidence of cardiovascular events since the new coronary pandemic. However, no direct evidence of adverse outcomes directly related to N95 mask wear has been reported. The purpose of this study is to identify the effects of prolonged N95 mask wear on the human cardiovascular and energy metabolic systems, which is important for optimizing the management of metabolic diseases in the COVID-19 pandemic.

2.2 Research Background

Since the beginning of the COVID-19 pandemic, N95 masks have been used extensively worldwide in everyday and medical scenarios [1]. To avoid the spread of COVID-19, wearers often wear them for long periods of time. Several studies have now reported a global surge in the incidence of cardiovascular events since the new coronary pandemic [2, 3]. However, no direct evidence of adverse outcomes directly related to N95 mask wear has been reported, and this study aims to clarify the effects of prolonged N95 mask wear on the human cardiovascular, energy metabolic system, which is important for optimizing the management of metabolic diseases in a COVID-19 pandemic.

2.3 Expected results of the study

The purpose of this study was to determine the effect of prolonged wear of N95 masks on human metabolism.

2.3.1 Known potential risks

Subjects in this study were required to wear an N95 mask for a prolonged period of time (14 consecutive hours) and to exercise at 40% of maximum oxygen consumption while wearing the mask, which may result in increased heart rate, respiratory rate and decreased oxygen saturation due to poor ventilation. There may be a potential risk for subjects with underlying medical conditions.

2.3.2 Known potential benefits

Each subject in this study will receive a full range of physical examinations and metabolic testing that may identify potential metabolic disorders. Subjects participating in this study will be diagnosed and instructed by a medical professional. Each subject will receive a full metabolic report at the end of the metabolic chamber testing.

2.3.3 Potential risk/benefit assessment

Since the beginning of the COVID-19 pandemic, N95 masks have been used extensively worldwide in everyday and medical scenarios. To avoid the spread of COVID-19, wearers often wear them for long periods of time, and although some researchers have found an increased risk of cardiovascular events and diabetes since the beginning of the pandemic, there is no direct evidence of adverse outcomes directly related to N95 mask wear. Also, during the course of this study, the investigators will evaluate the safety of the subjects. Therefore, the potential risks of this study are manageable and the potential benefits outweigh the potential risks.

3 Principal Investigator Information

3.1 Principal investigator's name, qualifications, contact information

Unit Ruijin Hospital, Shanghai Jiao Tong University

Name Prof. Weiqing Wang

Phone 021-64370045-671803

3.2 Key participants

Serial number	Name	Gender	Age	Title	Specialties	Whether GCP training	Role in the study (eg: PI,sub-I,CRC)
1	Wang Weiqing	Female	59	Chief Physician	Endocrine	Yes	PI
2	Sun Shouyue	Male	47	Chief Physician	Endocrine	Yes	Sub-I
3	Sun Yingkai	Male	36	Assistant Researcher	Endocrine	No	Sub-I
4	Pan Shijia	Female	26	Engineer	Endocrine	No	Sub-I

4 Purpose of the study

1. **Main purpose**.

   Evaluation of the effects of wearing N95 masks on glucose and lipid metabolism and cardiovascular system of subjects.

2. **Secondary purpose**.

Evaluation of the effect of wearing N95 masks on sleep quality.

Evaluation of the effect of wearing N95 masks on respiration and oxygen consumption.

Evaluation of the effect of wearing N95 masks on endocrine hormones.

5 Study Design

5.1 Overall design

The study was self-controlled and randomized. Each subject was placed in the control group (without mask) and the intervention group (with mask) in a randomized order, and the entire experiment was conducted in a human metabolic chamber to ensure the consistency of the external environment. Each subject's exercise, meal, and sleep activities were consistent throughout the 24-h period between the control and intervention groups, and the investigator accompanied the subjects throughout the experiment, checking and communicating with them outside the chamber every hour to ensure consistency of behavior between the control and intervention groups to reduce the influence of confounding factors.

5.2 Study endpoints

- Main evaluation indicators
  - ✓ Heart rate
  - ✓ Energy expenditure, glycolipid oxidation
  - ✓ Intravenous blood glucose
- Secondary evaluation indicators
  - ✓ Oxygen saturation
  - ✓ Respiratory rate
  - ✓ Blood pressure
  - ✓ Sleep Staging
  - ✓ Endocrine hormones
  - ✓ Blood lipids

5.3 Sample size

According to the statistical requirements, this study used self-control, two-sided test of variance, α=0.05, and with reference to our pre-experimental data, the sample size was calculated as 4% change in blood glucose on the control day at a level of 5.24 ± 0.24 mmol/L in subjects with 14 hours of mouthpiece intervention, with a sample size of 17.The test efficacy could reach 80%. The heart rate on the control day was 77.65 ± 3.35 and the heart rate on the intervention day was 81.83 ± 5.67 BPM, based on the average heart rate throughout the day.A sample size of 12 results in a test performance of 80%. With a sample size of 35, a test performance of more than 90% can be obtained for both heart rate and blood glucose. The final sample size was decided to be 30, taking into account the subject dropout rate.

6 Research Subjects

6.1 Selection Criteria

1. Healthy people aged 18 - 35 years
2. BMI between 18 - 28
3. Stable weight with less than ±3% change in the last three months
4. Healthy lifestyle habits, eating, sleeping and exercising regularly
5. No medication for the last three months
6. Fasting blood glucose < 6.1 mmol/L, HbA1c < 6.4%, no abnormal liver and kidney function during screening period
7. No caffeine and alcohol intake habits
8. No smoking habit
9. Able to sign informed consent forms independently

6.2 Exclusion Criteria

1. Patients with diabetes, hyperlipidemia, hypertension.
2. Other serious organic heart disease, such as congenital heart disease, rheumatic heart disease, hypertrophic or expanded cardiomyopathy, chronic congestive heart failure (NYHA ≥ Class III), or those who have received interventional therapy or used a cardiac stent, or have stage III hypertension (systolic blood pressure cannot be controlled below 160 mmHlg with three antihypertensive drugs)
3. People with hyper- or hypothyroidism.
4. Treated or untreated organ system tumors (other than localized cutaneous basal cell carcinoma) within the past 5 years, regardless of whether there is evidence of local recurrence or metastasis.
5. Gastrointestinal disorders affecting food digestion and absorption (e.g., severe abdominal feces, constipation, irritable bowel syndrome, inflammatory bowel disease, active peptic ulcer, acute cholecystitis, etc.): severe abdominal feces means watery stools twice a day or more for a period of days or more, severe constipation means 2 or fewer bowel movements per week with difficulty in defecation.
6. Persons with any mental illness or history of mental illness; persons with epilepsy or in anti-madness treatment with antidepressants.
7. Persons with severe infections, severe anemia, neutropenia.
8. Major surgery (except appendicitis, hernia surgery) within 1 year prior to screening.
9. History of previous bowel resection, or other GI surgery (e.g. cholecystectomy) within 1 year prior to screening, or other non-GI surgery within 6 months;
10. Use of weight control drugs (including diet pills), hormonal drugs such as corticosteroids (including oral, intramuscular or intravenous systemic, non-digestive or intra-articular) within 3 months prior to screening or planned during the trial.
11. Severe hepatic or renal dysfunction, or alanine aminotransferase (ALT) or aspartate aminotransferase (AST) ≥2.5× ULN, serum creatinine > UIN, glomerular filtration rate (eGFR) < 60mL/min/1.73m2 at screening (calculated using the MDRD formula)
12. Night shift or jet lag in the 1 month prior to screening

13. Antibiotic users in the 3 months prior to screening
14. Those who plan to use drugs (including traditional Chinese medicine and proprietary Chinese medicine) to change their weight within 3 months before screening or during the study period, or those whose weight fluctuates significantly (3 kg change in weight) within 3 months before screening.
15. Persons with a history of active substance abuse and/or alcoholism (> 40 g of alcohol/day for men and 20 g of alcohol/day for women, 40 g of alcohol is approximately 1000 mL of beer (6 proof), or 500 mL of red or yellow wine (12 proof), or 100 mL of white wine (50 proof))
16. Pregnant or lactating women
17. Participation in other clinical studies within 3 months prior to screening.
18. Any condition deemed by the investigator to be inappropriate for participation in this study.

6.3 Study Subject Recruitment

Study subjects were recruited from self-enrolled volunteers. Subjects will be enrolled after signing an informed consent form, being diagnosed by the study physician, and being evaluated according to the study protocol. Planned recruitment options include, but are not limited to, newspapers, the Internet, and posters.

6.4 Methodology of research subject assignment

This study is a randomized, self-controlled crossover trial. At screening, after obtaining informed consent, subjects will be randomly assigned a unique screening number by the investigator or his/her designee via the study center's SOP, and subjects will be administered the control and mask interventions in random order based on that number.

7 Research interventions

7.1 Intervention content

7.1.1 Intervention method

Subjects entered the control (no N95 mask) and intervention (wearing N95) phases in random order.

7.1.2 The items and number of clinical and laboratory tests to be performed

1. Informed Consent

The investigator explains all study procedures to the subject prior to screening and obtains voluntary informed consent from the subject (or their guardian or witness)

2. General Information

General information about the subjects was obtained during the screening period, including: date of birth, age, sex, and ethnicity.

3. Medical history

A history of significant medical conditions including diabetes, hyperlipidemia, and hypertension since before signing the informed consent form is recorded during the screening period. A history of allergies, drug abuse, alcohol abuse, and alcohol use will also be obtained during the screening period. The investigator will verify the inclusion/exclusion criteria based on the medical history.

4. Past medication history

During the screening period record the treatment used prior to signing the informed consent form, including prescription drugs, over-the-counter drugs, herbal medicines, other treatment history (including surgical history), etc. Record the start/end date of treatment, dose, frequency of treatment, route of administration or treatment method and indication, and that no medication can be taken from the time the informed consent form is signed until the end of the study. The investigator will verify the inclusion/exclusion criteria of the subjects according to their medication status.

5. Vital signs, physical examination

Vital signs include: blood pressure, respiration, temperature, and pulse. Physical examination includes: skin, mucous membranes, lymph nodes, head, neck, chest, abdomen, spinal cord/extremities, nervous system, etc.

Subjects' vital signs, physical examination, weight, waist circumference, hip circumference, visceral and subcutaneous fat area were recorded during screening period D0. Subjects' weight and body composition were recorded before and after the intervention period D2-D3. Vital signs were recorded dynamically during the intervention period D2-D3.

6. 12-lead electrocardiogram

Subjects' 12-lead ECG results were recorded during screening period D0.

7. Energy metabolism index

D2-D3 Visiting subjects were admitted to the metabolic chamber ward to measure energy metabolism indicators.

8. Maximum oxygen consumption measurement

Maximum oxygen consumption was measured using a Cosmed K5 respiratory energy meter in subjects who were screened into the group to determine the amount of exercise the subjects performed during the intervention period.

9. Laboratory tests

Subjects' laboratory test results were recorded at each visit during the screening and treatment periods.

Projects	Checking indicators
Blood Count	White blood cell (WBC), absolute neutrophil (NEUT#), lymphocyte percentage (LY%), monocyte percentage (MONO%), red blood cell count (RBC), hemoglobin (HGB), red blood cell pressure (HCT), platelet count (pLT) at screening period.<br>D0 Conduct.
Liver function 4 items	aspartate aminotransferase (AST), alanine aminotransferase (ALT), glutamate GGT, ALP. The screening phase was performed at D0
Renal function 1 item	Blood creatinine (Cr). Performed at screening period D0.
Blood lipids 4 items	High-density lipoprotein (HDL), low-density lipoprotein (LDL), total cholesterol (TC), triglycerides (TG) were performed at D2-D3.
Glucose metabolism index, insulin	Glycated hemoglobin (HbA1c) was performed at screening period D0. Fasting plasma glucose(FPG), fasting insulin (FINS) were performed in screening period D0, intervention period D2-D3.
Endocrine hormones	Free triiodothyronine (FT3), free thyroxine (FT4), thyrotropin (STSH1), blood cortisol (blood F), adrenocorticotropic hormone (ACTH), metanephrines (MN), noradrenaline (NMN)
Urea nitrogen	Urea (BUN) and urine volume were measured in the D2-D3.
Blood gas analysis, electroion, lactate	pH, partial pressure of carbon dioxide (pCO2), partial pressure of oxygen (pO2), sodium ion (cNa+), potassium ion (cK+),

	calcium ion (cCa+), chloride ion (cCl+), lactate (cLac), oxygen saturation (sO2), fraction of oxygen and hemoglobin (FO2Hb), fraction of carboxyhemoglobin (FCOHb), fraction of methemoglobin (FMetHb), reduced hemoglobin Protein fraction (FHHb) was performed in the D2-D3

10. Retention of biological samples

The collection and handling of biological samples (blood, urine) are described in Section 13.1 of the protocol. All biological samples collected for this study will be used only for the purposes of this study and will not be used by the investigator for any other testing. The investigator will not provide or sell the collected biological samples to other persons or companies.

11. Adherence evaluation

Screened subjects will wear an Actigraph activity meter during intervention period D2-D3, and each subject's activity in the crossover group will be recorded in full. Adherence = (actual daily activity/target activity) x 100%

7.2 Measures to reduce bias: randomization and control of confounding factors

The study was self-controlled and randomized. Each subject was placed in the control group (without mask) and the intervention group (with mask) in a randomized order, and the entire experiment was conducted in a human metabolic chamber to ensure the consistency of the external environment. Each subject's exercise, meal, and sleep activities were consistent throughout the 24-h period between the control and intervention groups, and the investigator accompanied the subjects throughout the experiment, checking and communicating with them outside the chamber every hour to ensure consistency of behavior between the control and intervention groups to reduce the influence of confounding factors.

7.3 Adherence judgment

Screening Period

Subjects who have signed informed consent will complete all screening tests during this period and the study physician will determine whether the subject meets the inclusion/exclusion criteria for this study protocol. After enrollment, the study physician will provide diet and exercise instructions to the subjects, and the subjects should follow a strict routine during the study period to ensure that they eat, exercise, and sleep consistently.

Balance period

Subjects were admitted to the study ward, maintained a regular routine according to the study plan, and faithfully recorded the time spent on each activity during the 24-hour period. A device was worn to detect the amount of exercise and dynamic blood glucose changes. Adherence was judged by the study physician based on his or her records.

Expectations

Subjects were housed in a human metabolic chamber, maintained a routine according to the study plan, and recorded the time spent in each activity over a 24-hour period. Exercise and dynamic blood glucose changes were measured with the device. Adherence was determined by the study physician based on their records.

7.4 Research Intervention Commitment

Adherence was determined by the investigator to be within the range of 90% - 110% of the measured activity level by completely recording the daily activity level and duration of activity.

7.5 Research Flow Chart

	Screening Period	Balance period [1]	Expectations	
	D0	D1	D2	D3
		(Each day is calculated from 8:00 - 7:59 the next day)		
**Basic Information**				
Informed Consent Form	✓			
General Information [2]	✓			
Medical history, past medical history	✓			
Inclusion/exclusion criteria [3]	✓			
Subjects were randomized [4]		✓		
Medication History	✓			
**Safety evaluation and general evaluation**				
Adherence evaluation	✓			
Measurement of maximum oxygen consumption	✓			
Height, weight	✓			
Vital signs [5]	✓			
Blood count [6]	✓			
Liver function 4 items [7]	✓			
Renal function 1 item [8]	✓			
Fasting blood sugar	✓			
HbA1c	✓			
12-lead electrocardiogram	✓			
Recording adverse events	✓			
**Equipment wear**				
Remove the mask [9]			✓	✓
Photovoltaic equipment [10]			✓	✓
Actigraph [11]		✓	✓	✓
Sports bracelet [12]		✓	✓	✓
Ambulatory blood glucose [13]		✓		
**Pre-entry testing**				
Inbody body composition measurement [14]		✓	✓	✓
DEXA Bone Densitometry			✓ [15]	✓ [16]
**Metabolic compartment testing**				
Postprandial metabolic testing [17]			✓	✓
Exercise metabolic testing [18]			✓	✓
Sleep metabolism testing [19]			✓	✓
Basal metabolic measurements [20]			✓	✓
**Laboratory testing** [21]				
Blood sugar			✓	✓

Insulin			✓	✓
Blood lipids			✓	✓
Alpha triplet			✓	✓

	Screening Period	Balance period [1]	Expect ations	
	D0	D1	D2	D3
	(Each day is calculated from 8:00 - 7:59 the next day)			
Cortisol			✓	✓
ACTH			✓	✓
NMN, MN			✓	✓
Urea nitrogen [22]			✓	✓
Lactic acid, blood gas analysis [23]			✓	✓
Blood Sample Retrieval [24]			✓	✓

1. Equilibration period: Subjects were required to finish dinner by 18:00 the day before the start of the equilibration period and to report to the study center by 18:30, wear the wearable device, and stay overnight in the general ward.
2. General information: including age, gender, BMI.
3. Inclusion/exclusion criteria: see 6.1 and 6.2 for details.
4. Subject randomization: each subject was assigned to the control (D2) - intervention (D3) period according to the subject's random assignment number in the D2 - D3 intervention period, respectively.
5. Vital signs: blood pressure, respiration, body temperature, pulse.
6. Blood count: white blood cell (WBC), absolute neutrophil (NEUT#), lymphocyte percentage (LY%), monocyte percentage (MON0%), red blood cell count (RBC), hemoglobin (HGB), red blood cell pressure (HCT), platelet count (PLT)
7. Blood biochemistry tests include liver and kidney function and lipid tests. Aspartate aminotransferase (AST), alanine aminotransferase (ALT), Y-glutamine transferase (GGT), alkaline phosphatase (ALP)
8. Kidney function 1: blood creatinine test
9. Wearing the mask: The N95 mask was started at 8:00 AM on the day of the intervention according to the random order of the subjects, and the nasal and cheek gaps were checked to ensure closure after wearing the mask
10. Equipment wear and removal: dry expected D2-D3 daily 7:50 a.m. before entering the cabin to replace the battery and wear, check the correct display of equipment data and enter the cabin, the next day 7:20 out of the cabin and remove
11. Actigraph wear and removal: The Actigraph activity detector was worn at 8:00 am during equilibrium D1 and intervention period D2-D3, in the right thigh, and could be removed at 7:30 am the following morning and re-worn by 8:00 am after the subject had completed personal clean-up (except after D3 exit).
12. Exercise bracelets: Exercise bracelets were worn at 8:00 am during equilibrium period D1 and intervention period D2-D3 and could be removed the following morning at 7:30 am and re-worn by 8:00 am after the subject completed personal clean-up (except after exiting the cabin in D3).
13. Ambulatory glucose: The ambulatory glucose probe and transmitter were worn and calibrated to fingertip glucose by 16:00 the day before the start of equilibration period D1 and again to fingertip glucose at 22:00 that evening. During equilibrium period D1 and intervention period D2-D3, they are calibrated with venous glucose at 8:00 and 22:00 each day, and removed upon completion of D3.
14. Inbody body composition measurements: measured at 7:30 AM daily during equilibrium period D1 and intervention period D2-D3. Fasting and urine evacuation are required before measurement
15. DEXA Bone Densitometry (pre-intervention): measured at 7:30AM prior to admission, fasting and urine evacuation required prior to measurement
16. DEXA Bone Densitometry (post-intervention): Subjects complete post-intervention DEXA measurement at 7:30 AM after discharge from the D3 intervention, with fasting and urine evacuation required prior to measurement

(continued)

	Screening Period	Balance period [1]	Expect ations	
	D0	D1	D2	D3
	(Each day is calculated from 8:00 - 7:59 the next day)			

17. Postprandial metabolic assay: It was performed three times a day, at 8:00 - 9:00, 13:00 - 14:00, and 17:00 - 18:00, during the dry period D2-D3, and the procedure for each assay was as follows.

    Pre-meal phase: 10 minutes in a sedentary position.

    Meal phase: end the meal within 30 minutes.

    Post-prandial phase: Continue to remain in a sedentary position after the meal until the end of the test.

18. Exercise metabolic testing: performed twice daily during intervention period D2-D3, at 11:10-12:20 and 15:40-16:50, respectively, to confirm the integrity of metabolic compartment, heart rate, pulse rate, respiratory rate, oxygen saturation and blood pressure data before each test. The testing process was as follows.

    Pre-exercise phase: remain in a sedentary position for 10 minutes, complete blood sample collection (lactate, blood gas) and fill out the Brog Fatigue Scale during this time period.

    Exercise phase: After meditation, set the cycling resistance according to the exercise volume calculated in advance (morning time period: 40% of maximum oxygen consumption; afternoon time period: 20% of maximum oxygen consumption) and maintain the speed at 60 rpm for 30 minutes, during which the Brog fatigue scale was filled in every 10 minutes.

    Post-exercise phase: After the exercise, return to a sedentary position for 30 minutes and fill in the Brog Fatigue Scale every 10 minutes.

19. Sleep metabolic testing: performed during interventional period D2-D3, once daily, from 21:10 to 6:00 each day, to confirm the integrity of the metabolic compartment, heart rate, pulse rate, respiratory rate, oxygen saturation, and blood pressure data before each test. The test procedure was as follows.

    Pre-sleep phase: Subjects complete pre-sleep food clean-up and exit the chamber to wear sleep EEG equipment and return to the chamber by 21:50.

    Sleep stage: 22:00 - 6:00 of the next day is the sleep time, subjects should stop other activities after 22:00, turn off the lights and start sleeping, if it is difficult to fall asleep, should keep lying still and try to fall asleep, other activities that affect sleep are prohibited (such as playing with cell phones, etc.).

    Wake up phase: The following day at 6:00, the lights were turned on and the subjects were woken up to enter the basal metabolism measurement phase.

20. Basal metabolic testing: performed once daily during intervention period D2-D3, from 6:00 a.m. to 7:00 p.m. the following day, after waking the subject at 6:00 a.m. and confirming that he or she remains awake while remaining in a sedentary state for 1 hour. The integrity of the metabolic compartment, heart rate, pulse rate, respiratory rate, oxygen saturation, and blood pressure data should be confirmed during the test.
21. Venous blood glucose, lipid and endocrine hormone tests were performed twice a day during intervention period D2-D3, before cabin entry (7:30-8:00) and before bedtime (21:10-22:00), respectively. Lipid measurements included: triglycerides (TG), total cholesterol (IC), low-density lipoprotein cholesterol (LDL-C), high-density lipoprotein cholesterol (HDL-C), and free fatty acids (FFA). Thyroid tests include: free triiodothyronine (FT3), free thyroxine (FT4), thyrotropin (STSH1), noradrenaline (MN), and norepinephrine (NMN). Samples will be sent to the Department of Internal Medicine or Laboratory Medicine at Ruijin Hospital for testing.
22. Urea nitrogen, urine routine: conducted in the dry period D2-D3, four times a day, collected from 8:00 - 12:30, 12:30 - 17:00, 17:00 - 21:30, respectively.Urine is measured at four time intervals from 21:30 - 8:00 the following day, and urine volume, urea nitrogen, and urine specific gravity are measured. During this period subjects should maintain adequate hydration throughout the day

23. The amount of water consumed should be greater than 1500 ml, and the amount of water consumed should be the same for each subject in the control and intervention states. The samples were sent to the testing department of Ruijin Hospital for testing.
24. Lactate and blood gas analyses were performed during the dry period D2-D3, four times a day, at 8:00, 11:00 (before exercise), 11:50 (after exercise) and 22:00, respectively. Parameters measured included: pH, partial pressure of carbon dioxide (pCO2), partial pressure of oxygen (pO2), sodium (cNa+), potassium (cK+), calcium (cCa+), chloride (cCl+), lactate (cLac), oxygen saturation (sO2), fraction of oxygen and hemoglobin (FO2Hb), fraction of carboxyhemoglobin (FCOHb), fraction of high iron hemoglobin fraction (FMetHb), and reduced hemoglobin fraction (FHHb). Samples were measured using a Radomite ABL90 blood gas analyzer. Blood samples were collected from D2-D3 during the intervention period, twice daily, at 8:00 and 22:00, respectively, for venous blood sampling. At each time point, 3 ml of serum (yellow tube) was collected from the subjects.

8 Study intervention termination and subject termination/withdrawal

8.1 Study intervention termination

(1) Reasons for full stoppage of the entire trial at multiple centers include and are not limited to.

- Researchers find serious safety concerns
- Significant program failures
- Discontinued for financial or management reasons
- Administrative authorities to withdraw the test

(2) Full suspension of the test can be temporary, but also permanent. Suspension of the test, the entire test should be retained for reference.

8.2 Subject termination/withdrawal

(1) Severe hypoxemia, hypoglycemia, hypotension caused by unconsciousness and other emergencies occurred

(2) Subjects fail to complete the requirements of the experiment according to the protocol

(3) Patient withdrawal of informed consent

(4) Sudden onset of menstruation during the trial in female patients

(5) The investigator deemed it necessary for the study subjects to terminate this study.

9 Evaluation of study outcomes

9.1 Primary and secondary efficacy evaluation indicators

(1) Main evaluation indicators

✓ Heart rate

✓ Metabolic rate

✓ Intravenous blood glucose

✓ Glycolipid oxidation amount

(2) Secondary evaluation indicators

✓ Oxygen saturation

✓ Respiratory rate

✓ Blood pressure

✓ Sleep Staging

✓ Endocrine hormones

✓ Blood lipids

9.2 Safety evaluation indicators

Adverse events, physical examination, vital signs, 12-lead electrocardiogram, laboratory tests (routine blood, urine, blood biochemistry)

9.3 Adverse Events and Serious Adverse Events

9.3.1 Adverse Event (AE) Definition

Adverse event: Any adverse medical event, whether or not causally related to the experimental intervention, between the time the subject signs informed consent and is enrolled in the trial and the last follow-up visit, is considered an adverse event

9.3.2 Serious Adverse Event (SAE) Definition

Serious Adverse Event: It means that any one of the following should be considered as a serious adverse event.

Death

Life threatening

Resulting in hospitalization or longer hospital stays

Permanent or severe disability or loss of function

Congenital anomalies or birth defects

Other medically significant events, which, although they would not have led to Article 15 above, if based on proper medical judgment, such events may

Such events may also be considered serious adverse events if they endanger the subject and require medical or surgical interventions to prevent the occurrence of Article 15.

9.3.3 Adverse Event Reporting

All AEs (whether related to the experimental intervention or not) must be fully documented from the time the informed consent form is signed until the end of the study.

9.3.4 Serious Adverse Event Reporting

For all serious adverse events occurring during a clinical study, whether reported for the first time or as a follow-up, the investigator must immediately complete and sign and date a Serious Adverse Event Report Form and report it immediately (no later than 24 hours after the SAE is known) to the sponsor or an authorized representative. The investigator should ensure that all required information is submitted within the above time frame. For all serious adverse events, it is the investigator's obligation to obtain and provide information to the sponsor within the reporting timeframes mentioned above. In general, a sufficiently detailed description of the adverse event should be included to allow for a thorough medical evaluation of the case and an independent assessment of the likelihood of causality. In addition, the investigator must provide information on other possible causes of the adverse event, such as combined medications and comorbidities. In the event of a subject's death, the investigator must submit a summary of autopsy results (if available) to the sponsor or its authorized representative as soon as possible. contact information for SAE reporting is detailed in the investigator's file provided to each center. The original SAE report form must be kept in a safe place by the center

10 Statistical Analysis

10.1 Analysis of data sets

(1) Full analysis set (FAS): is the ideal set of subjects that is as close as possible to the principle of intentional analysis (primary analysis to include all randomized subjects), which is derived from all randomized subjects with minimal and reasonable culling and contains all

Subjects who were randomized and had at least one complete control and mask intervention. Missing values for key variables were estimated using carry-forward to the nearest observation to the missing trial data, with the number of subjects in each group evaluating efficacy at the endpoint remaining the same as at the beginning of the trial.

(2) Protocol compliant set (PPS): as a subset of the FAS dataset for more adherent protocols: required to refer to the set of subjects who met inclusion criteria, did not meet exclusion criteria, and completed the treatment protocol, i.e., no serious violations of the protocol (including inclusion criteria) and good adherence for analysis (PP analysis)

(3) Safety Data Set (SS): Actual data from at least one intervention and control with documented safety indicators. No comparison of safety deficit tip values is required.

10.2 General Method

Detailed statistical methods will be provided in the statistical analysis plan. Statistical processing was performed by R (version 4.0 or higher). Pairwise t-tests were used for between-group comparisons of all-day dynamic data statistics. Laboratory tests before and after the intervention were tested for differences between groups using mixed linear models, and categorical indicators were tested using chi-square tests or exact probability methods (if chi-square tests were not applicable)

10.3 Analysis of primary and secondary study endpoints

(1) Analysis of primary study endpoints:

- Heart rate, energy metabolism, and glycolipid oxidation: pairwise t-tests were used for intergroup comparisons of all-day statistics
- Intravenous glucose: mixed linear model to test for differences between groups (Fixed effect: post-intervention glucose ~ subgroup + pre-intervention glucose.
  Random effect: Individual ID)

(2) Analysis of secondary study endpoints.

- Respiratory rate, blood pressure, sleep staging: paired t-test for intergroup comparison of all-day statistics
- Endocrine hormones, blood lipids: mixed linear model to test for differences between groups (Fixed effect: post-intervention values ~ subgroup + pre-intervention values; Random effect: individual ID)

10.4 Baseline descriptive analysis

Baseline information for both groups was recorded as descriptive statistics. Continuous variables are expressed as mean ± standard deviation, categorical variables are expressed as percentages, and skewed variables are expressed as medians.

11 Supporting Documents and Notes

11.1 Informed Consent

It is the responsibility of the investigator to obtain written informed consent from the subject prior to the start of any study procedures and to allow sufficient time to discuss any issues raised by the subject. Prior to enrollment in the study, the investigator will explain to each appropriate subject the nature and

purpose of the study and the benefits and risks of participating in the study. This is an investigator-initiated study and a sample informed consent form will be provided by the investigator. The final version of the informed consent form must be agreed to by the Ethics Committee, include all elements contained in the sample informed consent form, and be in language that is easily understood by the subject. The original informed consent form for each subject must be signed by the subject (or his or her guardian or witness) and by the study physician who absconded from the informed consent discussion and

The original informed consent form must be dated, remain in the institution's records, and be subject to inspection by a representative from the White regulatory agency, and the investigator provides a copy of their signed informed consent form to all subjects enrolled

11.2 Privacy Protection

It is the responsibility of the investigator to maintain the subject's anonymity. Subjects may be identified on case report forms or other documents only by uppercase letters, numbers, and/or codes, not by subject's name. The investigator must maintain the subject enrollment form with the subject's code, name and home address. The investigator must maintain strict confidentiality of documents that reveal the subject's identity.

11.3 Specimen and data collection and use

For the use and testing of specimens and information materials involved in the study, if third-party testing or storage is involved, the testing unit and address shall be specified: if a research institution/enterprise with foreign background is involved, it shall be submitted to the National Human Head Genetic Resources Office for approval; if the study is completed, the remaining specimens, visual materials and other data retained shall be used for the study of water after the consent of the study subjects. The location, manner, and duration of preservation of these specimens and data should be specified, and the informed consent should state that these specimens and data may be used for other relevant studies after the study is completed.

11.3.1 Experimental Process Management

The data sources for this study were mainly derived from human metabolic compartments and laboratory tests of serum/urine, which were administered as follows.

1. Human metabolic chamber testing: Subjects arrive at the study center by 7:30 a.m. each day of intervention period D2-D3 for equipment fitting and update.
   At 8:00 a.m., each subject was assigned a designated person as the "experiment managercomplete the maintenance of the cabin equipment and data collection. And establish
   Human Metabolic Chamber Experiment Record, which includes: subject ID, intervention type, randomization group, quality control report of experimental data, contingencies, etc.
2. Laboratory testing: The items that need to be sent to the laboratory for testing are as follows.

Stage	Sample (per subject)
D0	Routine blood sample x 1, blood biochemistry blood sample x 1, HbA1c x 1, venous blood glucose x 1
D1	None
D2	Intravenous plasma glucose x 2, insulin x 2, lipids x 2, metronomic x 2, cortisol x 2, ACTH x 2, urea nitrogen x<br>4, Lactic acid/blood gas x 4, NMN/MN x 2
D3	Intravenous plasma glucose x 2, insulin x 2, lipids x 2, metronomic x 2, cortisol x 2, ACTH x 2, urea nitrogen x<br>4, Lactic acid/blood gas x 4, NMN/MN x 2

After the specimens are collected, the samples to be sent by the commissioner to the laboratory for timely testing, sample storage and handling procedures are described in Annex 13.1, and the corresponding indicators are tested and statistically analyzed by the center's laboratory testing program. The test uses a unified view of the determination of anticoagulation blood collection tubes and other consumables.

11.3.2 Improve observation consistency measures

1. The personnel who participate in the observation and collection of clinical data should have a high level of professional knowledge and skills and be relatively fixed.

2. All experimental procedures and data cleaning are standardized and strict SOPs are established to reduce batch-to-batch variation

3. Pre-trial training should provide investigators with a thorough understanding and knowledge of the clinical trial protocol and the specific content of each indicator. The description of white sensation symptoms should be objective and should not induce or suggest: the objective indicators specified should be examined at the time and in the way specified in the protocol. Adverse reactions and unanticipated toxic effects should be observed and followed up. The case record form should not be left blank and should not be arbitrarily altered.

4. Information collars with significant deviations or outside the acceptable range were verified and the necessary explanations were provided by the investigator.

5. Each testing project must indicate the unit of measurement used.

6. Each clinical research unit should designate a person to regularly check the progress of clinical trials and carefully verify the information and records.

7. If necessary, the team leader will organize a clinical interim meeting to check the preliminary work, analyze the problems found during the clinical trial, and propose corrections.

8. The establishment of the clinical diagnosis and the assessment of the efficacy after treatment should be made by at least one attending physician and one resident, and the observation form should be completed by a resident or above.

11.3.3 Laboratory quality control requirements

Hospital laboratories should establish standard operating procedures and quality control procedures for the observation of experimental pointers. When the main indicators may be subjectively influenced, consistency testing is required, and effective measures should be taken to correct when there are large differences in test results or different ranges of normal reference values among the central laboratories.

11.3.4 Measures to Ensure Subject Adherence

1. We should work well with the subjects to make them understand the significance of the trial and have their families supervise the use of probiotics. For patients with poor efficacy and those who cannot use probiotics on time, follow-up should be strengthened.

2. Supervisors are appointed by the research unit to ensure that the rights and interests of the subjects in the clinical trials are protected, that the trial records and reports are accurate and complete, and that the trials follow the approved protocol, GCP and relevant regulations.

11.4 Data processing and record keeping

11.4.1 Data Collection and Management

1. Metabolic chamber dynamic data: This experiment establishes a complete database of the metabolic chamber for each subject, complete storage of raw data of all devices in the metabolic chamber, and establishes a standard cleaning process according to the sampling frequency of each device, and establishes a standard cleaning protocol for the treatment of extreme values, null values, and abnormal values. We also keep the raw data and processing documentation for each step of processing. All anomalies encountered by the researcher in the analysis are traced back to the most original data for verification and documented. The completed cleanup data are handed over to the statistical analysts for analysis in a standardized structure.

2. Laboratory test data: The laboratory data of this trial adopts the EDC system then establishes the eCRF. the data administrator establishes the corresponding eCRF according to the requirements of the trial, and the physician or clinical trial coordinator logs into the EDC system with the account and password for data entry. the investigator should record all relevant information of each subject in the trial in the CCRF in a timely and authentic manner. The data administrator and statistician will monitor the quality of the data entered in real time and issue questions in a timely manner. Problems or unexpected situations found during the entry process should be registered and reported in time so that the problems can be dealt with quickly. The system automatically issues queries at the same time of data entry, such as date, inclusion criteria, exclusion criteria, shedding, missing values, etc. Especially, the important indicators of statistical analysis are checked in detail using computer programs, and the researcher can directly answer the queries online, or the supervisor can download the data query form, and the

researcher will answer the questions in the query form in writing and sign before the data entry personnel describe the data revision and entry in a unified manner The data entry staff will then be unified to perform data revision and entry. For system queries, the researcher should answer the questions as soon as possible, and the data can be re-issued if necessary after answering the queries. After all data queries are resolved in the system, the data manager will export the "clean" data to the statistician, and the data will be reviewed by the lead researcher, statistical analyst, and data manager, and the data review resolution will be signed by the representatives of all parties involved in the meeting. After all parties have approved the data lock, the data manager performs the database lock and submits the locked data to the statistician for statistical analysis. Any changes to the data will require a signed consent form from the principal investigator, statistician and data manager before proceeding.

11.4.2 Publishing and data sharing conventions

All information regarding this study (not limited to the following documents: protocol, investigator's manual) must be kept strictly confidential. The investigator has the right to publish or distribute information or data related to the wood study.

11.4.3 Conflict of Interest Statement

None of the investigators or research associates participating in this study had any personal financial or non-financial interests or any direct or indirect obligations or responsibilities that conflicted with their job duties during this study.

12 Refereces

1. S. Esposito, N. Principi, C. C. Leung, and G. B. Migliori. Universal use of face masks for success against covid-19: evidence and implications for European Respiratory Journal, 55(6), 2020.
2. M. M. McDermott. The international pandemic of chronic cardiovascular disease. jama, 297(11):1253- 1255, 2007.
3. J. Wu, M. A. Mamas, M. Mohamed, C. S. Kwok, C. Roebuck, B. Humberstone, T. Denwood, T. Luescher,M. A. de Belder, J. E. Deanfield, et al. Place and causes of acute cardiovascular mortality during the covid19 pandemic: retrospective cohort study of 580, 972 deaths in england and wales, 2014 to 2020. medRxiv, 2020.

13 Annexes

13.1 Standard operating procedures for sample testing and delivery

I. Purpose

Standardize the conditions and processes for preserving, transporting, and receiving samples.

II. Simple process

Samples are collected in the Human Metabolic Chamber platform and are stored, divided and stored by the investigator. Samples to be sent for testing are sent to the appropriate laboratory within 30 minutes by a dedicated person. The sample receiving unit (each laboratory) is responsible for receiving the samples and completing the assay in a timely manner.

III. Acquisition and processing

(i) Blood samples

Collection: blood biochemistry, cortisol, methyl work, insulin with yellow tube; NMN/MN, HbA1c with dark purple tube; intravenous glucose with gray tube; routine blood with light purple tube; ACTH, blood gas/lactate with dark green tube.

Handling: Immediately after collection, gently turn the blood sample over 8-10 times and store it at 4°C in a timely manner, and notify someone to come and take the sample for testing within 30 minutes. If the sample cannot be sent for testing within the specified time, it should be centrifuged at 3000 rpm for 15 minutes at 4°C within 1 hour and then placed in the laboratory.

(ii) Urine samples

24-hour urine sample collection: ensure that the subject voids the urine before entering the cabin, and issue a urine collection jar to each subject at 08:00, and send the urine produced during each time period at 12:30, 17:00, 21:30 and 8:00 the next day, respectively.
