# Supplementary material for: Evaluation of Mask-Induced Cardiopulmonary Stress: A Randomized Crossover Trial
Source: JAMA Netw Open. 2023 Jun 9;6(6):e2317023. doi: 10.1001/jamanetworkopen.2023.17023 (PMC10257095; doi:10.1001/jamanetworkopen.2023.17023)
Supplement: Supplement 2. — eMethods. eFigure. Study Design eTable. Basal Characteristics of Participants eReference. [file jamanetwopen-e2317023-s002.pdf]

Supplementary Online Content

Bao R, Ning G, Sun Y, Pan S, Wang W. Evaluation of mask-induced cardiopulmonary stress: a randomized crossover trial. *JAMA Netw Open.* 2023;6(6):e2317023. doi:10.1001/jamanetworkopen.2023.17023

eMethods.
eFigure. Study Design
eTable. Basal Characteristics of Participants
eReference.

This supplementary material has been provided by the authors to give readers additional information about their work.

© 2023 Bao R et al. *JAMA Network Open.*

eMethods.

Ethical approval

This study protocol was registered as "Effect of N95 Face Masks on Human Metabolism: A Randomized Cross-Over Clinical Trial" at chictr.org.cn as ChiCTR2200057813.

Participants

Thirty-four healthy participants were recruited from 102 subjects who were interested in this study. After screening and exclusions, 30 participants completed this study and their basal characteristics were shown in **Supplementary Table 1**. Eligibility criteria included 18 ≤ age ≤ 35 years, 18 ≤ BMI ≤ 28 kg/m^2, Hb1Ac between 4.7 and 6.4%, fasting glucose between 3.9 and 6.1 mmol/L, resting heart rate between 60 and 100, and normal blood pressure profile. We excluded participants with shift work or jet lag within a month and during the experiment, medication use over the past three months, and diseases with abnormal clinical manifestations that need to be excluded, including but not limited to the nervous system, cardiovascular system, blood, lymphatic system, respiratory system, digestive system, urinary system, immune system, kidney, liver or metabolic disorders, or mental system diseases, breastfeeding or pregnant, insomnia, and contraindications for maximal oxygen uptake (VO2max) test. The participants were instructed not to perform strenuous physical exercises and not to consume alcohol or caffeine for 48 hours before the study. All participants provided written informed consent prior to participation in the trial.

Study design

The total design of this study was exhibited in **Supplementary Figure 1**. Each eligible subject underwent a randomized cross-over intervention. Entire studies were accomplished in the metabolic chamber, during which the calories of each meal and physical activity levels were rigorously controlled. Participants underwent standardized acclimatization on the first day. The subsequent two days were the intervention period,

© 2023 Bao R et al. *JAMA Network Open.*

where participants were randomized to receive interventions with and without the N95 mask (3M 9132, 3M, MN, USA). Daily activities during the intervention were strictly controlled according to the standardized schedule. During the sedentary behaviors, participants were allowed to watch TV and use the computer or smartphone. Participants were asked to do 30 minutes of exercise on their 40% VO2max level and 20% VO2max level by using an ergometer in the morning and afternoon, respectively. During the study, participants received an isocaloric, controlled nutrient diet consisting of 50% CHO, 20% protein, 30% fat, and a minimum of 1.5 liters of fluid per day.

Energy expenditure and substrates oxidation

To measure energy expenditure and substrates oxidation, participants resided in the metabolic chamber for the whole experiment period. The metabolic chamber is an airtight room with a volume of 30,000L each. The chamber is furnished with an adjustable bed, desk, chair, bicycle ergometer, wash basin, and toilet. With room temperature maintained at 25.0 ± 0.1°C, air in the chamber was pulled out at a fixed rate of 80 L/min. Concentrations of oxygen (O2) and carbon dioxide (CO2) in the sample air were measured by the Promethion (Model GA-06/FG-01) integrated instrumentation (Sable Systems International, Las Vegas, USA). The gas analyzer was calibrated weekly using standard gas for best performance. O2 consumption (VO2) and CO2 production (VCO2) were calculated by Henning method. To minimize the error of the metabolic chamber, we calibrated the accuracy by propane combustion. The precision of metabolic chamber was 99.3 ± 0.8% for O2 consumption and 100.0 ± 0.6% for CO2 production during the study. VO2 and VCO2 data produced by Henning's method were obtained at the frequency of 1 min. And then we aggregated these minute-based data into hourly-based for statistical comparison. Macronutrient oxidation and energy expenditure were calculated using the Weir equation with urinary nitrogen excretion. And urinary nitrogen was measured in 24h-urine (in four segments). All female participants performed respiratory chamber testing during the follicular or luteal phases of their menstrual cycle.

© 2023 Bao R et al. *JAMA Network Open.*

Continuous glucose monitoring

All participants were fitted with a continuous glucose monitoring (CGM) sensor and transmitter (Glunovo X1, Infinovo, Nantong, China) during the study period. The glucose-oxidase-based electrochemical sensor was inserted into subcutaneous tissue of the abdomen, followed by an initial warm-up period for 3 hours after sensor insertion. Sensor insertion began the day before the acclimatization day and there were two days of calibration before data collection during the intervention. According to manufacturer recommendations, the sensors were calibrated at least once every 12 hours by finger prick (FreeStyle Lite; Abbott Laboratories, Abbott Park, IL).

Maximal oxygen uptake

Prior to the study, each participant performed a maximal oxygen uptake (VO2max) test on the cycle ergometer (939E, Monark Ltd, Vansbro, Sweden). Oxygen consumption and carbon dioxide production were measured by a breath-by-breath portable gas analyzer (K5, COSMED, Rome, Italy). The test procedure started with 5 minutes warm-up at a workload of 0 W. Then, the workload was increased by 25W or 20W every minute for men or women, respectively, while participants maintained a pedaling rate of 60 rpm until exhaustion despite verbal encouragement. Participants met at least 2 of the following criteria were considered maximal: respiratory exchange ratio > 1.15, maximal heart rate > 90% of the predicted max (220-age), ratings of perceived exertion (RPE) ≥ 18, a plateau in VO2 despite an increasing workload. The exercise intensity was set at 40% VO2max (light intensity exercise) and 20% VO2max (very light intensity exercise) in this study, which represent light intensity exercise and very light intensity exercise, respectively.

Physiological parameters measure

Noninvasive blood pressure, 3-lead ECG, and peripheral pulse oximetry SpO2 were continuously monitored by a Cardiac Telemetry System (WEP-5204C, Nihon Kohden Co., Tokyo, Japan). Systolic and diastolic blood pressure (BP) were measured every 30

© 2023 Bao R et al. *JAMA Network Open.*

minutes during the study. Heart rate, respiration rate, and SpO2 were measured every second during the study. The physiological activity was monitored with the GT3X monitor (ActiGraph, Pensacola, FL, USA). The device quantifies motion in the three orthogonal axes, including vertical, anterior-posterior, and medial-lateral, called vector magnitude (VM). VM was calculated as the square root of the sum of the squares of activity counts from each of the three axes. GT3X monitor was placed on the participant's right mid-thigh line during the entire study, except during dual-energy X-ray absorptiometry (DXA) or shower. All GT3X devices were initialized at a sampling rate of 30 Hz and were downloaded using 60 s epochs and analyzed using ActiLife software (Version 6.13.4). Body weight and composition were measured separately by Inbody (Inbody 770, Inbody Co. Ltd., Seoul, Korea) and DXA (GE Lunar iDXA, GE Healthcare, Madison, WI, USA). Height was measured in centimeters using the ultrasonic instrument (OMRON HNH-318; OMRON Corporation, Kyoto, Japan).

Blood sampling

During the intervention, after placement of an indwelling cannula located in a forearm antecubital vein or a dorsal metacarpal vein, blood samples were obtained at four time points including: before and 14 hours after the masked intervention as well as before and after light intensity exercise. Blood was drawn directly into a BD Preset syringe (BD Preset, BD Diagnostics, Plymouth, UK) for immediate blood gas analysis (Radiometer ABL 90 Flex, Radiometer, Brønshøj, Denmark). The blood samples taken before and after exercise were only used for blood gas analysis. Calculations of arterial blood gas were made using custom MATLAB scripts proved by Dearlove.[1] In briefly, the "buffer line" was drawn through venous pH and HCO3- values with a gradient of –(8.2 + 9.2 * [Hb]. Then the line was shifted to left by a distance of 0.1 * (0.98 - SvO2). The intersection point between the second buffer line and the line given by the Henderson Hasselbalch equation in the Davenport diagram. Finally, the root-finding method fsolve on R was used to point when these two curves meet, which gives the arterial values for pH and HCO3-. Concurrent blood sample were drawn into

© 2023 Bao R et al. *JAMA Network Open.*

vacutainers (BD Vacutainer, BD, Eysins, Switzerland) for analysis of blood metabolites. Samples were centrifugated at 4°C and measured or stored at -80°C until further analysis. Plasma metanephrine (MN) and normetanephrine (NMN) were quantified by reverse-phase high-performance liquid chromatography (Agilent 1100 series, Santa Clara, CA, USA) with electrochemical detection (ESA-A Dionex Company, Chelmsford, MA, USA). Serum cortisol was examined using an Access Immunoassay System (Beckman Coulter Inc., Fullerton, CA, USA). Plasma ACTH levels were measured using an ELSA–ACTH immunoradiometric assay method (Cisbio Bioassays, Codolet, France). Serum levels of total cholesterol (TC), triglyceride (TG), high density lipoprotein (HDL), low density lipoprotein (LDL), and free fatty acid (FFA) were measured by enzymatic method using an autoanalyzer (Beckman AU5800; Beckman Coulter Diagnostics, USA). Serum levels of total T3, total T4, free T3, free T4, TSH, and insulin were determined with automated chemiluminescent immunoassays (ARCHITECT ci16200 analyzer, Abbott Laboratories, USA). Venous blood glucose (VBG) concentrations were measured using an autoanalyzer (Modular P800, Roche, Basel, Switzerland).

Visual Analogue Scales

Participants were required to rate their overall discomfort using Visual Analogue Scales (VAS). The questionnaire was administered hourly during the daytime and the next morning at 7:00 am.

Statistical analysis

Based on our preliminary data that daily mean heart rate was 85.7 ± 2.93 beats/min during unmasked condition and 87.5 ± 3.38 beats/min during masked condition. We calculated that 30 participants in each group would be needed to give this study 85% power to detect the difference of heart rate between unmasked and masked conditions at the alpha value of 0.05. Participants underwent a randomized cross-over intervention. The generation of random allocation sequence was based on the random-number table and the randomization was further stratified by gender. The characteristics of the

© 2023 Bao R et al. *JAMA Network Open.*

participants were summarized using descriptive statistics and raw mean (± SD) or median (interquartile range) were used to describe normally and non-normally distributed quantitative variables, respectively. The treatment effect of intervention between unmasked and masked conditions are reported as least squares mean (LSM) ± SEM, whereas the data for each condition are reported as raw mean ± SEM. Differences between groups were estimated using a linear mixed-effects model with observed parameters as dependent variable, treatment group as independent variable, sequence and time as covariates, the participants as a random effect. Where significant, post-hoc tests were performed using Bonferroni corrections. Statistical analyses were performed using R version 4.02.

© 2023 Bao R et al. *JAMA Network Open.*

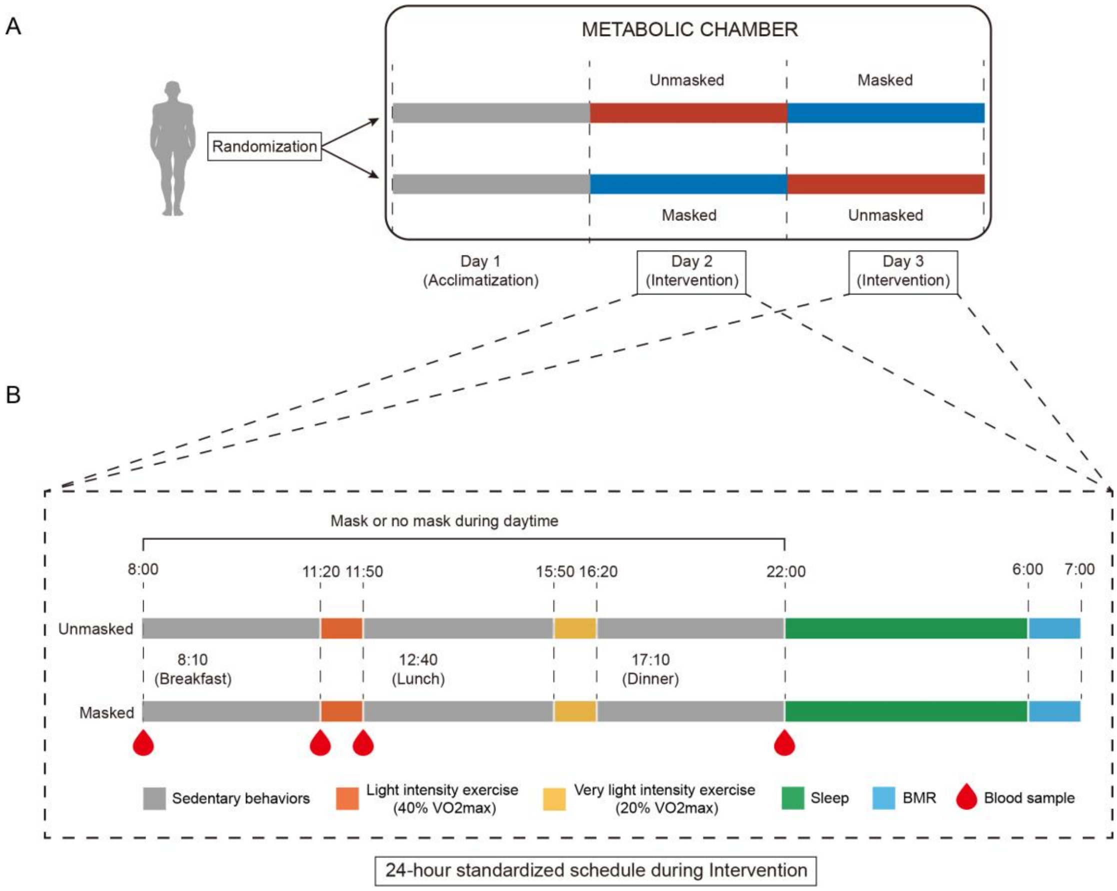

eFigure. Study Design. A: The overall study design over three days. B: The schedule of daily activity during the interventions.

© 2023 Bao R et al. *JAMA Network Open.*

eTable. Basal Characteristics of Participants

Characteristics	Unmask-first group	Mask-first group	p value[a]
No. (%) of participants (n = 30)	15 (50)	15 (50)	
Gender			
Women (%)	7 (46.7)	8 (53.3)	
Men (%)	8 (53.3)	7 (46.7)	
Age, mean (SD), years	26.8 (3.3)	25.2 (2.2)	0.14
Height, mean (SD), cm	167 (7.3)	165.1 (9)	0.53
Weight, mean (SD), kg	61.6 (8.6)	61.9 (8.6)	0.92
BMI, mean (SD), kg/m2	22 (2.1)	22.7 (2.5)	0.43
DXA			
Lean mass, mean (SD), kg	42.2 (7.4)	38.8 (7.4)	0.23
Fat, mean (SD), %	27.8 (5.5)	31.8 (8.7)	0.17
Neck circumferences, mean (SD), cm	34.1 (2.6)	35.2 (2.5)	0.26
Waist circumferences, mean (SD), cm	75 (6.5)	77.4 (7.4)	0.36
Hip circumferences, mean (SD), cm	94.6 (4.5)	96.5 (5.3)	0.29
Heart rate, mean (SD), beats/min	83.2 (17.3)	80.1 (14.8)	0.6
Respiratory rate, mean (SD), breaths/min	19.5 (2.1)	19.8 (2)	0.63
Systolic blood pressure, mean (SD), mmHg	111.5 (13)	111.3 (14.9)	0.97
Diastolic blood pressure, mean (SD), mmHg	73.8 (9.2)	72.9 (9.5)	0.8
Skin temperature, mean (SD), °C	36.2 (0.3)	36.4 (0.4)	0.1
Fitness level			
VO2 peak, mean (SD), mL/min	1981.8 (526.9)	1947.3 (431)	0.85
VO2 peak, mean (SD), mL/min/kg	32 (5.6)	31.6 (4.6)	0.8
Max watt completed, mean (SD), W	172.9 (42.8)	162.7 (34.9)	0.48
Fasting glucose, mean (SD), mmol/L	4.8 (0.4)	4.9 (0.3)	0.57
HbA1c, mean (SD), %	5.4 (0.3)	5.3 (0.2)	0.32

Abbreviation: BMI, Body mass index; DXA, Dual-energy X-ray absorptiometry.

SI conversion factors: to convert mmol/L to mg/dl, multiply by 18.0182.

[a] Differences between two groups were tested by independent samples t-test.

© 2023 Bao R et al. *JAMA Network Open.*

eReference.

1. Dearlove DJ, Faull OK, Rolls E, Clarke K, Cox PJ. Nutritional Ketoacidosis During Incremental Exercise in Healthy Athletes. Front Physiol. 2019;10:290. doi:10.3389/fphys.2019.00290

© 2023 Bao R et al. *JAMA Network Open.*
