# Supplementary material for: Evaluation of Mask-Induced Cardiopulmonary Stress: A Randomized Crossover Trial
Source: JAMA Netw Open. 2023 Jun 9;6(6):e2317023. doi: 10.1001/jamanetworkopen.2023.17023 (PMC10257095; doi:10.1001/jamanetworkopen.2023.17023)
Supplement: Supplement 3. — Data Sharing Statement [file jamanetwopen-e2317023-s003.pdf]

Data Sharing Statement

Bao. Evaluation of Mask-Induced Cardiopulmonary Stress. *JAMA Netw Open*. Published June 09, 2023. doi:10.1001/jamanetworkopen.2023.17023

Data
Data available: Yes
Data types: Deidentified participant data
How to access data: The data sets analyzed during the current study are available from the corresponding author upon reasonable request. Email:wqingw@shsmu.edu.cn
When available: With publication

Supporting Documents
Document types: Statistical/analytic code, Informed consent form
How to access documents: The statistical/analytic code and informed consent form during the current study are available from the corresponding author upon reasonable request. Email:wqingw@shsmu.edu.cn
When available: With publication

Additional Information
Who can access the data: Anyone requesting the data.
Types of analyses: For any purpose.
Mechanisms of data availability: With a signed data access agreement.
Any additional restrictions: None.
